# Supplementary material for: Modulation of Endocannabinoids by Caloric Restriction Is Conserved in Mice but Is Not Required for Protection from Acute Kidney Injury
Source: Int J Mol Sci. 2021 May 22;22(11):5485. doi: 10.3390/ijms22115485 (PMC8196977; doi:10.3390/ijms22115485)
Supplement: Supplementary file 1 [file ijms-22-05485-s001.zip › ijms-1212688-supplementary.pdf]

Table S1. Detailed patient characteristics.

	**CR_KCH**	**CR_LSP**	**Optifast**
Diet	CR	CR	CR
Number of subjects	19	6	8
Mean age (min/max)	68.2 (55/82)	52.4 (42.72/63.14)	49.88 (29/59)
Male (%)	73.7	66.66	37.5
Mean bodyweight at screening/kg (min/max)	79.95 (55.9/98.1)	90.93 (80.6/112.2)	129.91 (106.75/157.6)
Mean BMI (min/max)	27.38 (21/34.7)	28.98 (25.2/33.5)	42.21 (34.8/47.2)
Mean Δ weight (min/max)	−2.78 (−5.6/0.6)	−2.18 (−4/−0.6)	−22.48 (−33.4/−13.9)

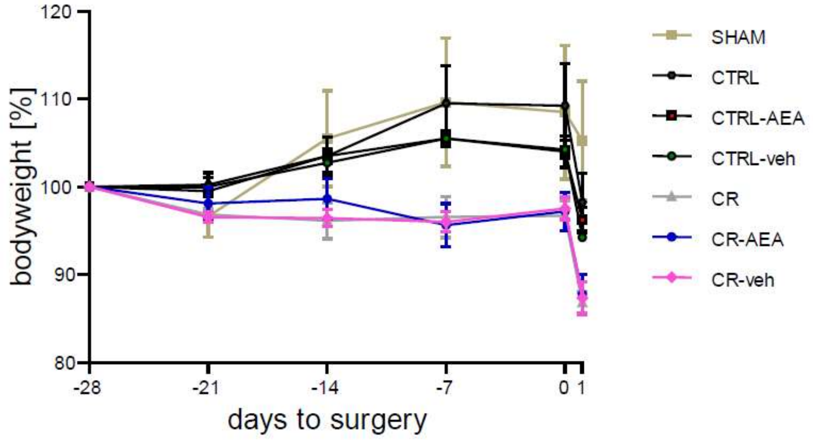

Fig. S1

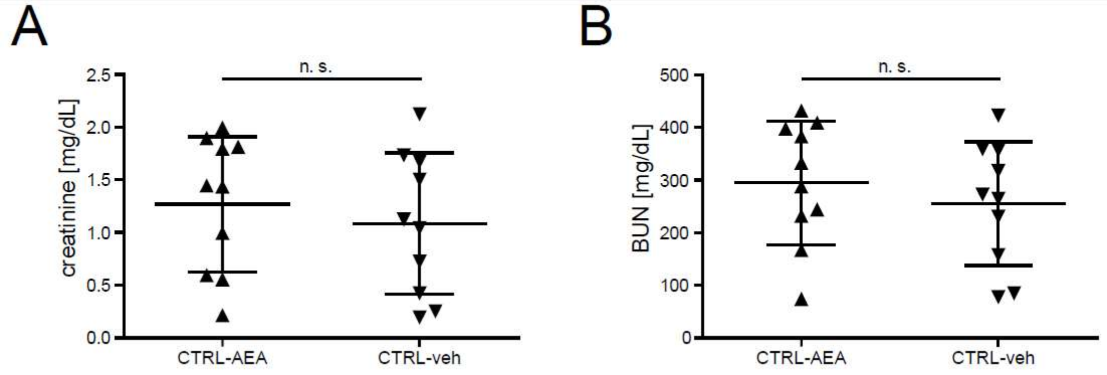

Fig. S2

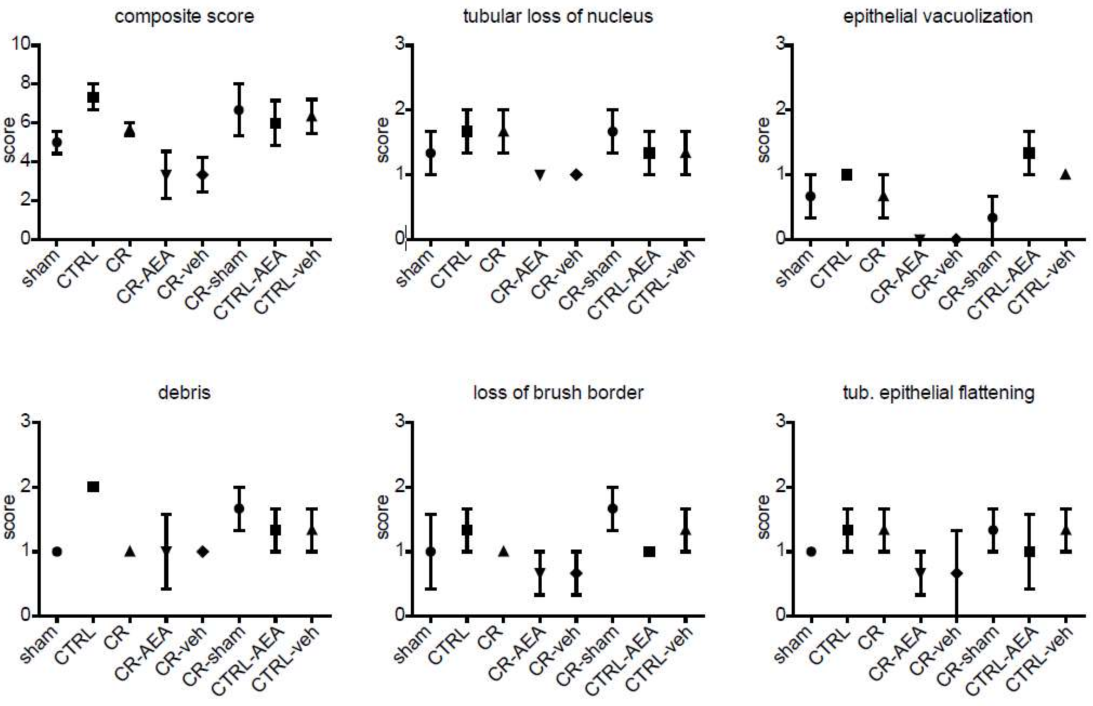
composite score
tubular loss of nucleus
epithelial vacuolization
debris
loss of brush border
tub. epithelial flattening
score
sham
CTRL
CR
CR-AEA
CR-veh
CR-sham
CTRL-AEA
CTRL-veh

Fig. S3

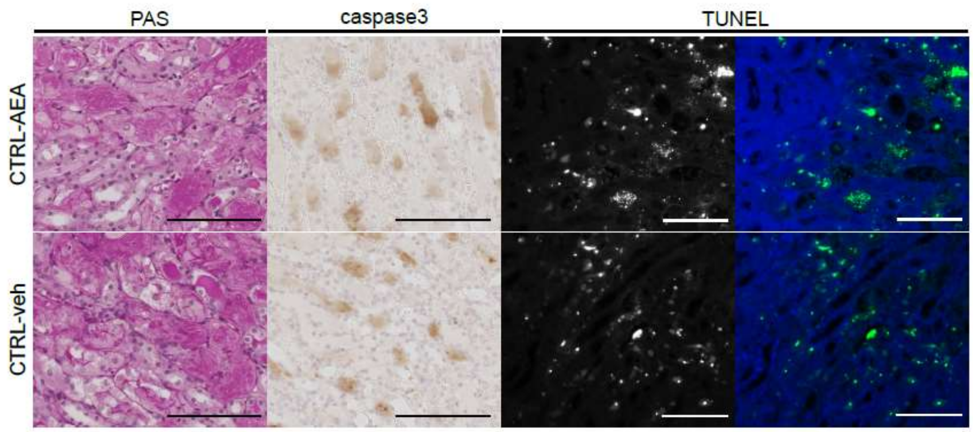
PAS
caspase3
TUNEL
CTRL-AEA
CTRL-veh

Fig. S4
